# Local social vulnerability as a predictor for cancer-related mortality among US counties

**DOI:** 10.1093/oncolo/oyae251

**Published:** 2024-09-14

**Authors:** Raymond Uduba, Divya Rath, Richard Gillum

**Affiliations:** Renaissance School of Medicine at Stony Brook, Long Island, New York, United States; Howard University College of Medicine, Washington, DC, United States; Howard University College of Medicine, Washington, DC, United States

## Abstract

This letter to the editor comments on the report by Chen et al that examined county-level social vulnerability index and age adjusted cancer mortality rates using linear regression models.

The recent report by Chen et al examined county-level social vulnerability index (SVI) and age-adjusted cancer mortality rates (AAMR) using linear regression models.^1^ They reported that counties in the highest quartile of SVI had higher AAMRs compared with those in the least vulnerable quartile. This was true for each of the 5 cancers accounting for most deaths. In reading this paper, we noted an apparent discrepancy in Figure 1. According to the figure legend, the map labeled “A” shows AAMR, whereas the map labeled “B” shows SVI. However, the legend next to the map “A” is for SVI, and the legend next to map “B” is for AAMR. We would appreciate the authors and editors resolving this possible error for the benefit of readers.

The report might also benefit from disclosure of which of the biennial updates of SVI was chosen for this analysis. Given the choice of deaths from 2013-2019, the 2016 SVI update (mid-period) would have been a reasonable choice. Or the 2014, 2016, and 2018 updates could have been combined. It would have been helpful to indicate that the purpose of SVI was not to predict health status but to indicate a community’s likely “ability to prevent human suffering and financial loss in the event of disaster.”^2^ A further question is the validity of the exclusion of the minority status item to create a modified SVI for this analysis. Since the authors apparently retained the item, “Speaks English less than well,” this item likely would still retain much of the information in minority status, ie, much more prevalent among Hispanics, the largest US minority, and other immigrant populations. This could bias results for Hispanics.

## References

[1] Chen KY , Blackford AL, Ramy S, Gupta A, Qasim Hussaini SM. Local social vulnerability as a predictor for cancer-related mortality among US counties. Oncologist. 2023;28(9):e835-e838. 10.1093/oncolo/oyad176 37335883 PMC10485383

[2] CDC/ATSDR Social Vulnerability Index 2016 update. Published June 13, 2020. Accessed July 17, 2023. https://www.atsdr.cdc.gov/placeandhealth/svi/documentation/SVI_documentation_2016.html

